# Hierarchical Chemotaxonomic Differentiation in Cannabis Chemovars Using Quantitative HPLC Cannabinoid Profiling and Multivariate Chemometrics

**DOI:** 10.3390/plants15071077

**Published:** 2026-04-01

**Authors:** Amonrat Mayong, Tanee Sreewongchai, Sasithorn Limsuwan, Natthasit Tansakul

**Affiliations:** 1 Agricultural Research and Technology Transfer Center, Faculty of Agriculture, Kasetsart University, Bangkok 10900, Thailand; amonrat.may@ku.th; 2 Special Research Incubator Unit for Cannabis-Hemp and Phytochemicals in Veterinary Medicine, Faculty of Veterinary Medicine, Kasetsart University, Bangkok 10900, Thailand; taneesree@gmail.com (T.S.); sasithorn.limsu@ku.th (S.L.); 3 Department of Agronomy, Faculty of Agriculture, Kasetsart University, Bangkok 10900, Thailand; 4 Institute of Food Research and Product Development, Kasetsart University, Bangkok 10900, Thailand; 5 Department of Pharmacology, Faculty of Veterinary Medicine, Kasetsart University, Bangkok 10900, Thailand

**Keywords:** *Cannabis sativa* L., cannabinoids, chemometrics, chemovar, HPLC, variety

## Abstract

The chemotaxonomic classification of *Cannabis sativa* L. has historically relied on the Δ^9^-tetrahydrocannabinol (THC) to cannabidiol (CBD) ratio, yielding canonical chemotypes I, II, and III. However, this binary framework overlooks the chemical diversity contributed by the minor cannabinoids. High-performance liquid chromatography (HPLC) following the AOAC Official Method 2018.10 was employed to quantify nine cannabinoids (THCA, THC, CBDA, CBD, CBGA, CBG, CBC, CBDV, and CBN) across 36 commercially and medicinally relevant cannabis varieties. Quantitative profiling revealed substantial phytochemical heterogeneity, with total THC ranging from 0.41% to 15.64% and total CBD ranging from 0.09% to 12.32% (w/w). Unsupervised principal component analysis (PCA) demonstrated that the first two principal components explained 62.7% of the total variance. PC1 (37.6%) captured the THCA–CBDA polarity axis, while PC2 (25.1%) was dominated by minor cannabinoids (CBC; loading 0.417), CBGA (0.314), and CBG (0.258). Supervised partial least squares discriminant analysis (PLS-DA) using only the nine cannabinoid variables achieved 94.2% cross-validated accuracy and 100% test-set accuracy in predicting the chemotype class, with CBC identified as the third most discriminatory variable (variable importance in projection, VIP = 1.34). Hierarchical clustering resolved three principal clades and further subdivided THC-dominant accessions into CBC-enriched (Sour Diesel, Cinderella Jack) and CBGA-enriched (Mother Gorilla, Auto Lemon Kix) subclusters. A multivariate “metabolic coordinate” system based on PC1/PC2 scores is proposed as a quantitative and reproducible alternative to the traditional Type I/II/III and sativa/indica nomenclatures. This study introduces an empirically grounded framework for variety authentication, quality control, and enhanced precision breeding in the rapidly growing medicinal cannabis sector, for both human and veterinary applications.

## 1. Introduction

The chemotaxonomic classification of *Cannabis sativa* L. has historically relied on the relative abundances of Δ^9^-tetrahydrocannabinol (THC) and cannabidiol (CBD), giving rise to canonical chemotype designations: Type I (THC-dominant), Type II (intermediate, mixed THC/CBD), and Type III (CBD-dominant) [1,2]. This framework, originally established through genetic segregation studies and subsequently corroborated by targeted metabolite profiling, has proven to be remarkably durable and continues to inform regulatory, agricultural, and clinical contexts. However, the cannabinoid biosynthetic pathway yields a considerably greater chemical diversity than the two primary metabolites alone. Cannabigerolic acid (CBGA) serves as the central branch-point precursor, from which all downstream cannabinoids are derived via the action of competing oxidocyclase enzymes: tetrahydrocannabinolic acid synthase (THCAS), cannabidiolic acid synthase (CBDAS), and cannabichromenic acid synthase (CBCAS) [3,4]. Subsequent decarboxylation, oxidative degradation, and side-chain variations (e.g., propyl vs. pentyl alkyl groups) further expanded the phytochemical repertoire to include cannabigerol (CBG), cannabichromene (CBC), cannabinol (CBN), and varin-class cannabinoids, such as cannabidivarin (CBDV) [5].

Despite growing recognition of their pharmacological relevance, such as CBG for anti-inflammatory and neuroprotective activities, CBC for antidepressant-like effects, and CBDV for anticonvulsant potential, the chemotaxonomic contribution of these minor cannabinoids remains poorly integrated into formal classification frameworks [6,7]. Most large-scale chemovar surveys continue to prioritize THC and CBD quantification, treating minor metabolites as incidental rather than informative variables [8,9]. Consequently, it remains unresolved whether targeted cannabinoid fingerprints alone, independent of terpene data, retain sufficient discriminatory resolution to describe intraspecific metabolic structures.

From a plant metabolic perspective, cannabinoid diversity can be interpreted as the differential allocation of metabolic flux from the CBGA branch toward competing oxidocyclase pathways. Such allocation patterns may reflect the underlying genetic regulation, selective breeding history, and pathway-level enzyme competition. Framing chemotype differentiation within this biosynthetic context offers an opportunity to reinterpret chemovar structure as a manifestation of intraspecific secondary metabolic plasticity rather than purely categorical classification.

To capture this diversity, the present study examined 36 genetically and commercially distinct *Cannabis sativa* varieties, encompassing THC-dominant (Type I), intermediate (Type II), and CBD-dominant (Type III) chemotypes. The panel included stabilized high-CBD breeding lines (Charlotte’s Angel Types 1–4), widely cultivated THC-rich varieties (e.g., Sour Diesel, Super Lemon Haze, Girl Scout Cookies), hybrid commercial varieties (e.g., Green Gelato, Wedding Crasher), and regionally adapted accessions (e.g., Hang Kra Rog Phu Phan). Rather than assessing within-variety variability, the design was structured to capture the broad intraspecific metabolic diversity across independent germplasms representing distinct breeding histories.

In this study, high-performance liquid chromatography (HPLC) was employed in accordance with the validated AOAC Official Method 2018.10 [10] to generate quantitative fingerprints of nine cannabinoids including acidic precursors, neutral decarboxylated forms, degradation products, and varin analogs across a diverse panel of 36 commercially and medicinally relevant cannabis varieties. These data were subsequently investigated using a tiered multivariate strategy designed to progressively resolve the latent chemical structure. First, principal component analysis (PCA) was applied as an unsupervised dimensionality reduction technique to determine whether intrinsic variance within the cannabinoid dataset enables chemotypic groupings to emerge independently of predefined classification labels. Second, partial least squares discriminant analysis (PLS-DA) was implemented as a supervised classification model to evaluate whether cannabinoid fingerprints could predict chemovar class membership and identify the specific metabolites driving intergroup discrimination. Third, hierarchical clustering analysis was performed and visualized as a heat map to reveal coordinated abundance patterns and assess biosynthetic coherence among varieties sharing nominal chemotype designations.

By integrating quantitative cannabinoid profiling with unsupervised, supervised, and clustering-based analytical frameworks, this study aimed to: (i) comprehensively characterize the phytochemical diversity of major and minor cannabinoids across chemo typically distinct varieties; (ii) determine the relative contributions of individual cannabinoids to chemotaxonomic variance; and (iii) evaluate the discriminatory power of focused cannabinoid panels for variety authentication and quality control. The resulting multivariate models provide an empirically grounded framework for cannabis chemotaxonomy that acknowledges both the primacy of the THCA/CBDA biosynthetic branch point and the chemotaxonomic value of the broader cannabinoid repertoire.

## 2. Results

### 2.1. Quantitative Profiling of Nine Cannabinoids

A total of 36 *Cannabis sativa* L. varieties were subjected to quantitative HPLC analysis. The validated HPLC–DAD method demonstrated excellent linearity over the concentration range of 0.01–0.4 mg/L (R^2^ ≥ 0.999), consistent with previously reported validation of cannabinoid quantification using the Vanquish™ platform [11]. These concentrations represent matrix-equivalent values calculated after applying the appropriate extraction and dilution factors to the measured extract solutions, thereby reflecting the final cannabinoid levels in the plant material rather than the concentrations in the injected extract. External calibration curves were constructed using mixed cannabinoid standards over the range of 0.25–10.0 µg/mL, corresponding to the analytical working range of the instrument for extract solutions prior to conversion to plant-matrix concentrations. All analytes exhibited strong linear responses (R^2^ > 0.99) across this calibration interval, confirming the suitability of this method for the quantitative determination of cannabinoids in diverse cannabis varieties.

Nine cannabinoids, including the acidic precursors (THCA, CBDA, CBGA), their neutral decarboxylated forms (THC, CBD, CBG), and the minor constituents (CBC, CBDV, and CBN), were unambiguously identified and quantified. The complete dataset for all 36 varieties is presented in Table S1 (Supplementary Materials).

Varieties such as El Patron, Blue Gelato, Super Lemon Haze, Godfather, and Sugar Bomb Punch exhibited THCA concentrations ranging from 13.15% to 15.90% (w/w), with the corresponding total THC (THCA × 0.877 + THC) reaching 14.65–15.64%. In these accessions, the CBDA remained consistently below 0.05%, and the total CBD did not exceed 0.24%. Notably, within this group, the CBGA pool varied considerably; Mother Gorilla accumulated 0.91% CBGA, whereas El Patron contained only 0.15% CBGA. This six-fold variation indicates differential metabolic flux at the CBGA branch point, independent of the downstream THCA synthase efficiency. All Charlotte’s Angel variants (Types 1–4) and Black Jack displayed reciprocal chemical signatures. The CBDA concentrations ranged from 6.52% to 11.28%, and the total CBD reached 7.66–12.32%. The THCA remained below 0.56% and the total THC did not exceed 0.72%.

Green Gelato and Hang Kra Rog Phu Phan exhibited balanced accumulation of THCA and CBDA. Green Gelato contained 6.16% THCA and 8.28% CBDA, yielding a total THC:total CBD ratio of 0.79. The Hang Kra Rog Phu Phan contained 1.54% THCA and 1.15% CBDA with a ratio of 1.73. CBGA, the universal precursor, was detected in all samples; however, its concentration ranged from 0.05% (Hang Kra Rog Phu Phan) to 0.91% (Mother Gorilla). Elevated CBGA was not strictly associated with either THC or CBD dominance; rather, it appeared in varieties where downstream conversion may be rate-limiting. For instance, Auto Lemon Kix contained 0.51% CBGA, despite being THC-dominant, suggesting a partial bottleneck at the THCA synthase step. CBG, the decarboxylated product of CBGA, showed a similar distribution. The highest CBG levels were found in Godfather (0.16%) and Blue Gelato (0.13%), both with high THC levels.

Sour Diesel and Cinderella Jack, both classified as THC-dominant, contained 0.09% and 0.12% CBC, respectively, which is approximately twice the average of other THC-dominant varieties. This enrichment indicated genotype-specific expression or allelic variation in CBCA synthase. CBDV, a propyl-side-chain cannabinoid, was detected above the LOQ in 13 of the 36 varieties, with concentrations ranging from 0.03% to 0.05%. CBDV was present in both THC-dominant (e.g., O. G. Kush, Sugar Bomb Punch) and CBD-dominant (Charlotte’s Angel, Black Jack) backgrounds. CBN, an oxidative degradation product of THC, was detected in trace levels (<0.04%) in approximately one-third of the samples.

### 2.2. Principal Component Analysis (PCA)

**Figure 1:**
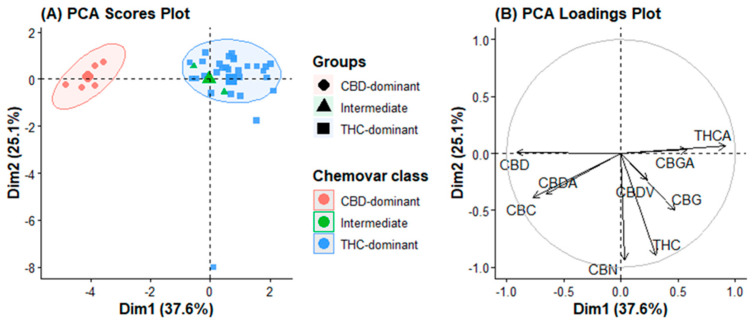
Principal component analysis (PCA) of cannabis chemovars based on cannabinoid composition. (**A**) Scores plot showing separation of CBD-dominant, intermediate, and THC-dominant chemovars. (**B**) Loadings plot illustrates the contribution of individual cannabinoids to sample discrimination.

PCA was performed on the covariance matrix of the nine quantified variables to visualize the intrinsic variance structure of the cannabinoid dataset without any prior classification. The first two principal components captured 37.6% (PC1) and 25.1% (PC2) of the total variance, cumulatively explaining 62.7% of the dataset’s information content (Figure 1A). PC3 and PC4 explained an additional 12.3% and 8.1% of the variance, respectively, cumulatively reaching 83.1% (Figure S2, Supplementary Materials). Examination of PC3 loadings revealed no additional chemotaxonomic patterns beyond those captured by PC1 and PC2; therefore, subsequent analyses focused on the first two principal components. A scree plot was generated to determine the optimal number of principal components to retain (Figure S2), confirming that the inflection point (elbow) occurs after PC2, indicating that the first two components capture the dominant variance structure.

The score plot projected along PC1 revealed a clear uninterrupted continuum. THC-dominant varieties (e.g., El Patron, Blue Gelato, Super Lemon Haze, Godfather) occupied the negative PC1 space, whereas CBD-dominant varieties (all Charlotte’s Angel variants, Black Jack) clustered tightly in the positive PC1 space. Intermediate varieties (Green Gelato and Hang Kra Rog Phu Phan) were positioned near the origin, bridging the two poles.

Examination of the loading plot (Figure 1B) revealed the metabolites responsible for this separation. THCA exhibited the highest absolute loading on PC1 (0.496), followed closely by CBD (0.493), and CBDA (0.358). The nearly equal but opposite contributions of THCA and CBD/CBDA confirm that PC1 is a quantitative readout of metabolic competition at the CBGA branch point. A single latent variable captures the essence of the classical type I, II, or III chemotype system.

The secondary variance along PC2 was dominated by the CBC (loading 0.417), CBGA (0.314), and CBG (0.258). This axis functions orthogonally to the major polarity axis. Varieties with exceptionally high CBC, Sour Diesel, and Cinderella Jack, were projected into the positive PC2 region, while high-CBGA accessions (Mother Gorilla, Auto Lemon Kix) occupied the negative PC2 region. Importantly, THC-dominant varieties did not form a single cluster in the PC2 space; instead, they were dispersed according to their minor cannabinoid composition.

No natural breaks or discrete clusters were observed in the PCA score plot. Even for the CBD-dominant varieties, the most biochemically homogeneous group formed a dense but continuous cloud rather than a sharply bounded cluster. This observation challenges the conceptualization of cannabis chemotypes as discrete categories, and instead supports a metabolic continuum model in which any segregation is an arbitrary division imposed on a fundamentally continuous distribution.

### 2.3. Partial Least Squares Discriminant Analysis (PLS-DA)

While PCA provides an unsupervised overview, supervised PLS-DA was employed to assess whether the nine-cannabinoid fingerprint could predict predefined chemotype labels (Types I, II, and III) and identify the most discriminatory metabolites.

**Figure 2:**
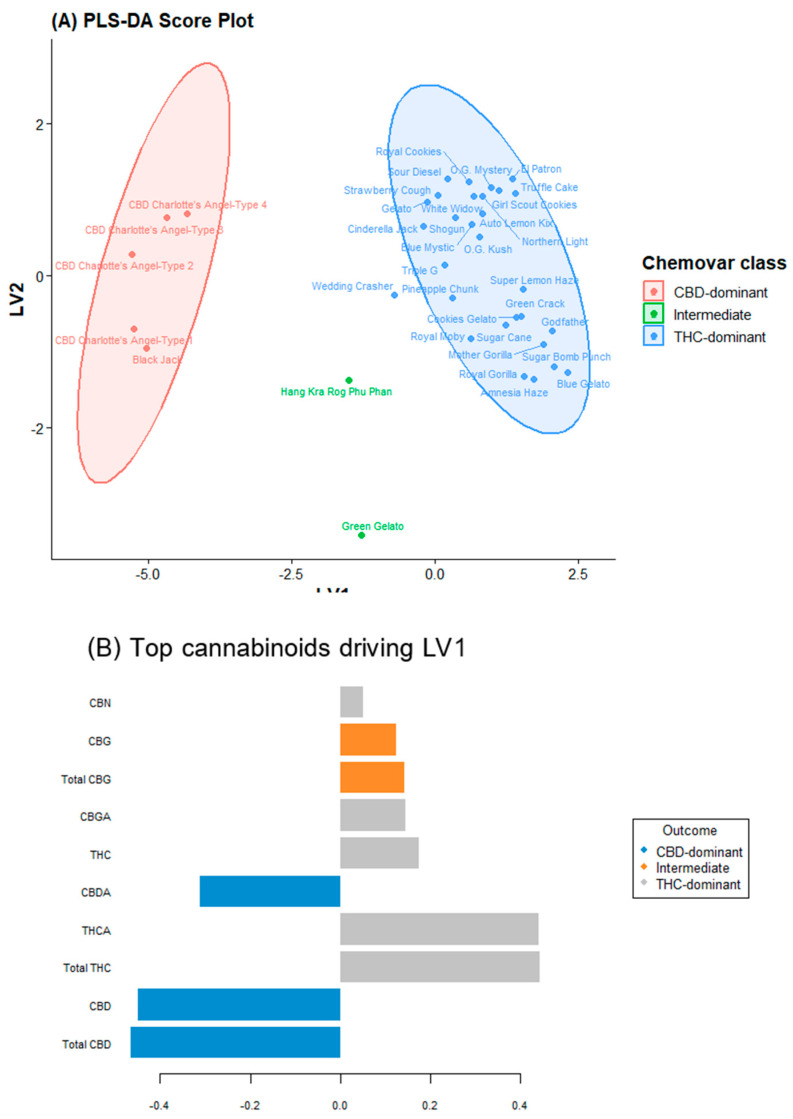
Chemometric discrimination and biomarker ranking of cannabis chemovars based on cannabinoid profiles. (**A**) Partial least squares–discriminant analysis (PLS-DA) score plot illustrating supervised classification of cannabis chemovars based on quantitative cannabinoid composition. (**B**) Bar plot of major cannabinoids contributing to LV1 discrimination.

The PLS-DA model was calibrated using a training set comprising 70% of the samples and validated using leave-one-out cross validation. The optimal model utilized six latent variables, yielding a cross-validated accuracy of 94.2% and Q^2^ value of 0.81, indicating excellent predictive power and no overfitting. An independent test set (the remaining 30% of the samples) was predicted with 100% accuracy, confirming the robustness of the cannabinoid-only classifier. The PLS-DA score plot (Figure 2A) demonstrated markedly enhanced separation compared to PCA.

The intermediate varieties were positioned precisely between the two dominant clusters along LV1, providing chemometric confirmation of their co-dominant synthase expression. Loading: Hierarchical importance of metabolites. The loading profile for LV1 (Figure 2B) recapitulated the PCA findings; THCA and CBDA exerted the strongest positive and negative influences, respectively. The secondary loadings along LV2 were dominated by CBGA, CBC, and CBG. Notably, the loading coefficient of the CBC was nearly as high as that of the major cannabinoids. VIP scores identified four cannabinoids with VIP > 1.0: THCA (1.82), CBDA (1.79), CBC (1.34), and CBGA (1.21).

The PLS-DA model achieved 94.2% cross-validated accuracy and 100% test-set accuracy. Given the limited number of intermediate samples (*n* = 2), permutation testing (*n* = 1000) was conducted to assess overfitting. The original model showed 100% training accuracy, while permuted models averaged 86.6% (max 97.2%). No permutation reached 100% accuracy (*p* < 0.001), confirming statistical significance.

The two intermediate varieties, Green Gelato (THC:CBD = 0.79) and Hang Kra Rog Phu Phan (1.73) positioned between THC- and CBD-dominant clusters in the score plot (Figure 2A), confirming their transitional status.

Class-wise metrics, presented in Table S2 (Supplementary Materials), show excellent: THC-dominant class (Type I): sensitivity = 1.000, specificity = 0.833, precision = 0.800, F1-score = 0.889. Intermediate class (Type II): specificity = 1.000, precision = 1.000, sensitivity = 0.500, F1-score = 0.667. CBD-dominant class (Type III): perfect scores across all metrics. These results confirm strong discriminatory power despite the intermediate class’s low sensitivity due to small sample size.

### 2.4. Hierarchical Clustering and Heatmap Visualization

**Figure 3:**
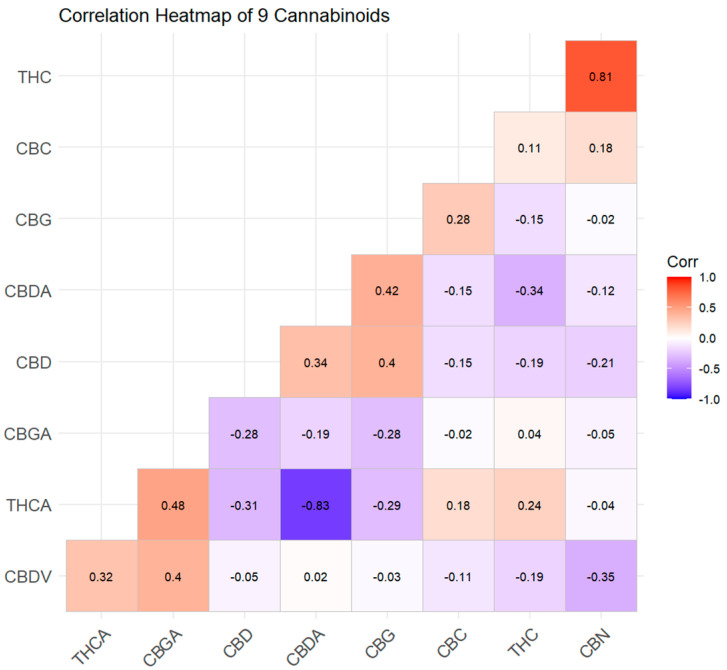
Correlation heatmap of nine cannabinoids quantified across 36 cannabis varieties. Color scale represents Pearson correlation coefficients (red: positive correlation; blue: negative correlation).

To visualize coordinated abundance patterns across both varieties and cannabinoids, hierarchical clustering was performed using Ward’s method with Euclidean distance, and the results are displayed as a color-scaled heatmap (Figure 3).

**Figure 4:**
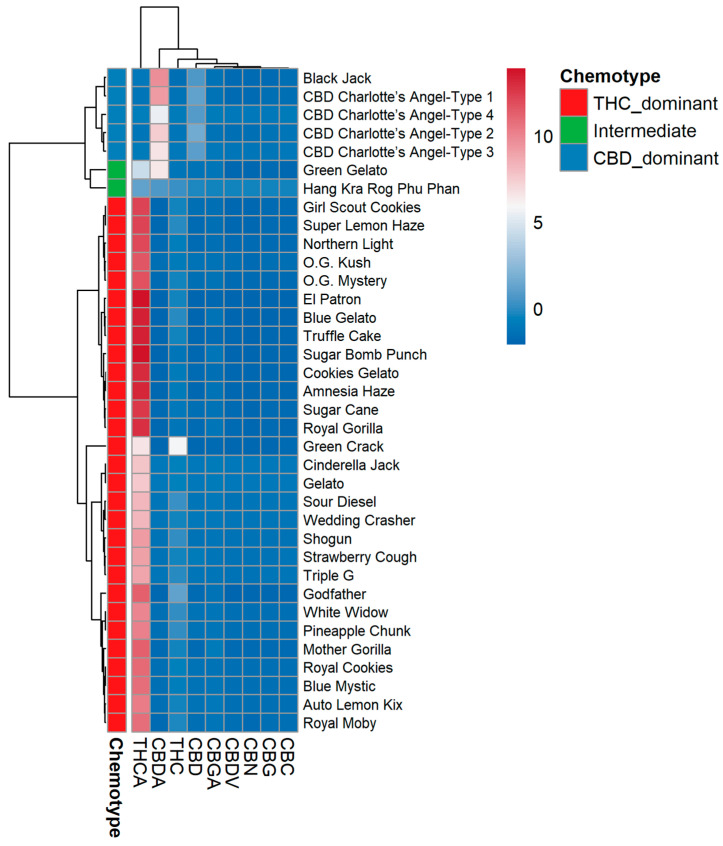
Hierarchical clustering heatmap of 36 cannabis varieties (rows) and nine cannabinoids (columns). Color scale represents row-centered abundance (blue = low, red = high). Variety dendrogram (**left**) shows three clades: Clade I (CBD-dominant), Clade II (intermediate), and Clade III (THC-dominant) subdivided into CBC-enriched and CBGA-enriched subclades. Cannabinoid dendrogram (**top**) reveals three clusters: A (THCA, THC, CBN), B (CBDA, CBD), and C (CBGA, CBG, CBC).

The variety dendrogram (Figure 4) resolved three principal clades. Clade I contained all CBD-dominant varieties (Charlotte’s Angel Types 1–4, Black Jack) united by high CBDA/CBD and low THCA/THC. Clade II contained intermediate varieties (Green Gelato and Hang Kra Rog Phu Phan) and a few THC-dominant accessions with atypically high CBGA or CBC. Clade III comprised the majority of THC-dominant varieties, but this clade was further subdivided into two distinct subclades: one enriched for CBC (Sour Diesel, Cinderella Jack), and one enriched for CBGA (Mother Gorilla, Auto Lemon Kix). This substructure is invisible in the conventional THC:CBD ratio classification.

The cannabinoid dendrogram revealed three distinct clusters of co-regulated metabolites:

1. Cluster A (THCA–THC–CBN): These three compounds formed a tight cluster, reflecting their direct biosynthetic relationship (THCA→THC→CBN). The co-clustering of CBN with THCA/THC, despite its very low absolute concentration, demonstrated that even degradation products carry phylogenetic signals.
2. Cluster B (CBDA–CBD): Isolated as a separate branch, confirming the independent regulation of the CBD pathway.
3. Cluster C (CBGA–CBG–CBC): This cluster grouped the three “branch-point” metabolites. Importantly, CBGA was not consistently associated with either the THCA or CBDA cluster; its variable positioning in the heatmap—sometimes linked to THCA and sometimes to CBDA—depended on the variety’s dominant synthase. This visualizes the biochemical role of CBGA as a universal but uncommitted precursor, the downstream fate of which is determined by the relative expression of THCAS versus CBDAS.

The heatmap visually validated the trends observed in the quantitative and PCA analyses, specifically confirming that the four Charlotte’s Angel “Types” are chemically indistinguishable. It also highlighted clear metabolic branching: Mother Gorilla and Auto Lemon Kix formed a unique CBGA-rich subgroup within the THC-dominant clade, whereas Sour Diesel and Cinderella Jack clustered into a distinct CBC-rich subgroup. Interestingly, the absence of any variety resulted in high levels of both CBC and CBGA simultaneously.

## 3. Discussion

This study demonstrates that minor cannabinoids, specifically CBC, CBGA, and CBG, possess discriminatory power comparable to and orthogonal to that of THC and CBD. Three independent multivariate methods support this conclusion: PCA revealed that CBC and CBGA dominate the secondary variance axis (PC2, 25.1% of total variance); PLS-DA identified CBC as the third most influential variable (VIP = 1.34), with a loading coefficient nearly as high as that of the major cannabinoids, reinforcing its status as a valuable chemotaxonomic marker rather than a mere trace constituent; and hierarchical clustering grouped CBC and CBGA into a distinct biosynthetic module independent of the THCA/CBDA dichotomy. The VIP scores confirmed that four cannabinoids exceeded the significance threshold: THCA (1.82), CBDA (1.79), CBC (1.34), and CBGA (1.21), demonstrating that the inclusion of minor cannabinoids is not redundant, but contributes unique discriminatory information unattainable from major cannabinoids alone.

The historical focus on forensic distinction between drug- and fiber-type cannabis prioritized THC and CBD quantification, with minor cannabinoids treated as incidental [12,13]. The present data refute the assumption that compounds present at lower concentrations lack chemotaxonomic value. However, the present data challenge this view: CBC, despite its low abundance (0.09–0.12%), exhibited high discriminatory power (VIP = 1.34), demonstrating that chemotaxonomic value is determined by genetic variance rather than absolute concentration [14]. This finding extends the work of Cerrato et al. [14], who identified unknown cannabinoids correlating with THC- or CBD-dominant strains by demonstrating that CBC and CBGA are the primary known constituents responsible for this discrimination. This dataset revealed a layered metabolic architecture. At the highest level, the competitive allocation of CBGA between THCA synthase and CBDA synthase defines primary chemotypic polarity, with THCA and CBDA exerting the strongest positive and negative influences, respectively, confirming that the primary discriminant is the allocation of CBGA flux. This primary polarity explained 78% of the total cannabinoid variance. At the secondary level, variety-specific efficiencies of precursor conversion, side-chain alkyl chain length, and post-harvest degradation generated a continuous spectrum of minor cannabinoid profiles that could not be predicted from the THC:CBD ratio alone.

### 3.1. Practical Sufficiency of Cannabinoid Fingerprints in Quality Control

A persistent question is whether reliable chemovar identification requires terpene profiling. Terpenes contribute to the entourage effect and can discriminate closely related varieties that share similar cannabinoid profiles [15,16]. While terpene profiling was beyond the scope of the present study, which focused on evaluating the chemotaxonomic utility of cannabinoid-only fingerprints, we acknowledge that the inclusion of terpenes would provide additional resolution for distinguishing closely related cultivars. Future studies integrating both phytochemical classes are warranted to develop a hierarchical chemotaxonomic framework. However, terpene analysis by GC-MS requires specialized instrumentation and is sensitive to postharvest handling [17]. Many quality control laboratories possess HPLC-UV capability but not GC-MS. Our PLS-DA model, built exclusively from nine HPLC-amenable cannabinoids, achieved 94.2% cross-validated accuracy and 100% test-set accuracy, which are comparable to published models incorporating both cannabinoids and terpenes [9,18,19]. For regulatory compliance, mislabeling detection, and pharmacopoeial monographs, a validated cannabinoid-only fingerprint is therefore sufficient.

The PLS-DA score plot demonstrated markedly enhanced separation compared to PCA. CBD-dominant varieties formed an exceptionally compact cluster, reflecting the genetic and metabolic canalization achieved by back-crossing the CBD-rich germplasm into a uniform background. This was exemplified by the four Charlotte’s Angel accessions, which—despite being labelled as distinct “Types”—showed nearly identical cannabinoid profiles, indicating successful breeding stabilization for high-CBD production. In contrast, the THC-dominant varieties exhibited broad, elongated dispersion along the first latent variable. This dispersion cannot be attributed to analytical errors or environmental variations because all plants were cultivated under identical controlled conditions. Instead, it reflects genuine genetic heterogeneity within the high-THC germplasm pool, a consequence of decades of informal polygenic selection focused almost exclusively on THC potency, which inadvertently preserved diverse minor-cannabinoid backgrounds.

We recommend that international pharmacopoeias expand the required cannabinoid test panels beyond THC and CBD to include CBC, CBGA, and CBG. The incremental analytical cost is minimal, as these compounds are resolved in most published HPLC methods [20], whereas the gain in discriminatory power is substantial.

### 3.2. Cannabis Chemotypes Exist on a Metabolic Continuum

The cannabis research community operates under two incompatible paradigms. The genetic paradigm recognizes accessions along a complex admixture continuum with no clear boundaries [21,22]. The regulatory paradigm imposes arbitrary thresholds that create discrete legal categories that lack biological basis. Our findings align with previous genomic studies demonstrating that Cannabis populations exist along a continuum rather than as discrete clusters [23]. Vergara et al. [23] demonstrated that commercial Cannabis exhibits continuous chemical and genetic diversity across lineages. Romero et al. [24] further emphasized that chemotype variation results from complex domestication history and metabolic pathway regulation. Our PCA results demonstrated that chemical phenotypes exhibit the same continuity as genotypes, with the score plot showing seamless gradation from extreme THC-dominant to extreme CBD-dominant chemotypes, with no natural breaks at regulatory thresholds. This continuity extends to minor cannabinoids and CBC and CBGA enrichment are quantitative rather than qualitative traits.

The mutually exclusive enrichment of CBC versus CBGA within the THC-dominant clade suggests two distinct high-THC chemovar subtypes, a level of resolution previously attainable only by whole-genome sequencing [25]. This dispersion within the broad “Type I” category indicates the existence of substantial and chemically meaningful substructures. From a biosynthetic perspective, CBCA synthase directly competes with THCA and CBDA synthases for CBGA [6]. Unlike THCAS and CBDAS, whose genes reside in a complex locus [25], CBCAS is independently regulated [26]. The observation that no variety displayed high levels of both CBC and CBGA simultaneously suggests a metabolic trade-off or specific channeling mechanism, where the plant’s precursor resources are directed toward one specific cannabinoid pathway at the expense of the other.

High-CBC accessions (Sour Diesel, Cinderella Jack) may therefore carry allelic variants that enhance CBCAS expression without compromising THCAS activity, a trait of interest for breeding programs targeting CBC’s emerging anti-inflammatory and neuroprotective properties of CBC [27]. Intermediate varieties, such as Green Gelato and Hang Kra Rog Phu Phan, represent true hybrid chemotypes in which neither synthase dominates the precursor pool.

We propose a conceptual shift toward multivariate, continuous chemotaxonomy using “metabolic coordinates”—PC1 and PC2 scores quantifying position on the THCA–CBDA polarity axis and minor-cannabinoid enrichment pattern. These two variables explained >60% of the total chemical variance and provided a quantitative, reproducible chemovar signature free from arbitrary cutoffs, preserving information distinguishing CBC-enriched from CBGA-enriched Type I varieties.

### 3.3. Breeding and Clinical Implications

The identification of two THC-dominant sub-clusters has direct breeding consequences. Breeders targeting CBC-rich medicinal cannabis should be selected from the CBC-enriched subclade (Sour Diesel, Cinderella Jack), whereas those aiming to maximize CBG content should focus on accessions with elevated CBGA pools (Mother Gorilla, Auto Lemon Kix). The near-identical profiles of the four Charlotte’s Angel Types raise conservation concerns: if distinct accessions are chemically indistinguishable, genetic diversity within CBD-dominant breeding pools may be dangerously narrow, increasing vulnerability to pathogens. Germplasm repositories should perform comprehensive metabolomic fingerprinting to identify and conserve the distinct accessions.

The finding that the THC:CBD ratio explains only 37.6% of the total chemical variance has implications for personalized cannabinoid therapy. In both human and veterinary medical contexts, it is crucial to understand that a formulation with a 1:1 ratio of THC to CBD should not be assumed to have pharmacological properties equivalent to those of other formulations with varying concentrations of CBC, CBGA, and CBG. Given emerging evidence that minor cannabinoids modulate THC psychoactivity [28], contribute to anti-inflammatory synergy [29] and neuroprotective effects [30], and possess independent therapeutic effects [31,32], we advocate full cannabinoid panel labeling of all medicinal cannabis products. Notably, CBDV was detected in 13 of the 36 varieties across both THC-dominant (O. G. Kush, Sugar Bomb Punch) and CBD-dominant (Charlotte’s Angel, Black Jack) backgrounds, indicating that the varin biosynthetic pathway is inherited independently of the pentyl-cannabinoid pathway. CBN, detected at trace levels (<0.04%) in approximately one-third of the samples, did not correlate with chemotype class but was more frequent in samples with higher absolute THC content, consistent with its formation during post-harvest storage.

### 3.4. Limitations and Future Directions

This study has several limitations. First, rare chemotypes (CBG-dominant Type IV and cannabinoid-free Type V) were not represented. Although dedicated Type IV (CBG-dominant) chemotypes were absent, several accessions exhibited high CBGA levels. Mother Gorilla (total CBG = 0.88%), Sugar Bomb Punch (0.80%), and Blue Gelato (0.72%) making them promising breeding material for future CBG-enriched varieties. Future studies should expand the sample panel to include dedicated Type IV and Type V varieties to further validate and refine the chemotaxonomic model proposed herein.

Second, a single growing environment and harvest time point controlled for environmental variance but could not assess marker stability across conditions. The observation that CBG concentration did not strictly parallel CBGA concentration suggests that decarboxylation kinetics or postharvest handling may influence the final neutral CBG content, warranting multi-environment field trials to establish genotype-by-environment interactions. Third, the terpenes were not quantified. While cannabinoid-only models are suitable for many applications, terpene integration provides a finer resolution. The ideal chemotaxonomic system is hierarchical: cannabinoids define primary metabolic polarity, terpenes define aromatic subtypes, and rare metabolites define unique signatures. Finally, the assumption that chemical differences translate to therapeutic differences requires testing in controlled human or animal trials comparing chemovars matched for THC and CBD, but differing in CBC or CBGA content.

## 4. Materials and Methods

### 4.1. Plant Material

A total of 36 cannabis cultivars (*Cannabis sativa* L.) were obtained from the Breeding and Research Unit, Faculty of Agriculture, Kasetsart University, Bangkok, Thailand, and were used for cannabinoid profiling in this study. The collection comprised 35 improved varieties, originally sourced from international seed bank companies, and one Thai landrace cultivar, “Hang Kra Rog Phu Phan.” All cultivars were cultivated in a controlled indoor system to ensure standardized growth conditions and high-quality biomass production. Due to limited plant material availability, a single representative plant per cultivar was analyzed. However, each sample was extracted and analyzed in triplicate (technical replicates) to ensure analytical precision and reproducibility. After harvest, inflorescences were dried at 25–30 °C with 50–55% relative humidity for 7 days in a dark, well-ventilated room until the moisture content was below 10%. Dried samples were pulverized using a laboratory mill (Retsch GM 200, Retsch GmbH, Hann, Germany) at 5000 rpm for 30 s, and the powder was passed through a 1 mm mesh screen to ensure uniform particle size. The ground material was stored in vacuum-sealed bags at −20 °C in the dark prior to chemical extraction and analysis.

All plant materials were obtained and handled in compliance with Thai narcotics regulations (Ministry of Public Health Notification No. 456/2564) under institutional approval from the Cannabis, Hemp, and Kratom Policy Committee, Kasetsart University (Approval No. MHESI 6401.22/208, dated 29 March 2023).

### 4.2. Chemicals and Reagents

Certified reference standards for THCA, THC, CBDA, CBD, CBGA, CBG, CBC, CBDV, and CBN (1.0 mg/mL in methanol or acetonitrile) were purchased from Cerilliant Corporation (Round Rock, TX, USA). HPLC-grade methanol, acetonitrile, ammonium formate, and formic acid (98%) were obtained from Sigma-Aldrich (St. Louis, MO, USA). Water was purified to 18.2 MΩ·cm using a Barnstead Smart2Pure system (Thermo Scientific, Waltham, MA, USA).

### 4.3. Sample Preparation and Extraction

Dried, ground inflorescences (200 ± 5 mg) were accurately weighed into 50 mL amber centrifuge tubes and extracted with 25 mL of 80% (*v*/*v*) aqueous methanol. Extraction was performed by sonication (Ultrasonic Bath, Isolab, Germany) at room temperature for 15 min, with vortex mixing every 5 min. The extract was centrifuged at 4500× *g* for 5 min (Eppendorf 5810 R, Eppendorf SE, Hamburg, Germany), and the supernatant was filtered through a 0.22 μm PTFE syringe filter (Phenomenex, Torrance, CA, USA) into an amber HPLC vial. All samples were extracted in triplicates.

### 4.4. Cannabinoid Quantification and Method Validation

Cannabinoids were analyzed using a Vanquish HPLC system equipped with a diode array detector (DAD) and an Accucore C18 column (150 × 4.6 mm, 2.6 µm; Thermo Scientific, Waltham, MA, USA) maintained at 40 °C. Chromatographic separation was achieved at a flow rate of 1.5 mL/min using mobile phase gradients of (A) 0.1% formic acid in water/acetonitrile (85:15, *v*/*v*) and (B) 0.05% formic acid in methanol/acetonitrile (64:36, *v*/*v*). The gradient program was as follows: 60% B (0–0.5 min), 65% B (8.5 min), 70% B (11.2 min), 95% B (13.0 min), and 98% B (14.0–16.0 min), followed by re-equilibration at 60% B for 22.0 min. The injection volume was 5 µL and the detection wavelength was set at 230 nm. Data acquisition and processing were performed using the Chromeleon software (Thermo Scientific). The method was validated according to the AOAC Official Method 2018.10. Linearity was established using a six-point calibration curve (0.25–10.0 µg/mL) with R^2^ > 0.990. The limits of detection (LOD) and quantification (LOQ) were 0.01% and 0.03% (w/w), respectively, expressed as mass of analyte per mass of dried plant material, based on 3σ and 10σ criteria. Recovery studies were performed by spiking blank plant matrix with known concentrations of cannabinoid standards, yielding recoveries ranging from 95% to 102% for all analytes. Peak identities were confirmed by UV spectral matching. A representative chromatogram of mixed cannabinoid standards, demonstrating the separation efficiency of the validated HPLC method (Figure S1). The total THC, Total CBD and Total CBG content were calculated using the decarboxylation correction factor (0.877):

Total THC = THC + (THCA × 0.877)

Total CBD = CBD + (CBDA × 0.877)

Total CBG = CBG + (CBGA × 0.877)

### 4.5. Multivariate Statistical Analysis

All statistical analyses were conducted using R version 4.3.1 (R Foundation for Statistical Computing, Vienna, Austria) within the RStudio environment (Posit, Boston, MA, USA). Principal component analysis (PCA) was performed using the FactoMineR package on mean-centered and unit-variance-scaled cannabinoid data to explore the intrinsic variance structure. The variable loadings and sample scores for the first two principal components were visualized using a biplot. Partial least squares discriminant analysis (PLS-DA) was performed using the mixOmics package to evaluate chemotype separation. The dataset was randomly partitioned into training (70%) and testing (30%) subsets. The optimal number of latent components was determined using 10-fold cross-validation. Model performance was assessed using classification accuracy, Q2 statistics, and the area under the receiver operating characteristic curve (AUC). Variable Importance in Projection (VIP) scores were calculated to identify the metabolites that contributed most strongly to class discrimination. Hierarchical clustering was performed using Euclidean distance matrices and Ward’s minimum variance linkage method. Heatmaps with corresponding dendrograms were generated using the pheatmap package to visualize the coordinated abundance patterns across varieties and metabolites.

## 5. Conclusions

This study provides an empirically grounded reassessment of cannabis chemotaxonomy using quantitative profiling. The findings demonstrate that minor cannabinoids, particularly CBC, CBGA, and CBG, possess discriminatory power comparable to THC and CBD, defining a secondary axis of chemical variation that enables subtype discrimination within the THC-dominant germplasm. A validated cannabinoid-only analytical panel classification accuracy confirmed that comprehensive cannabinoid fingerprints may be sufficient for regulatory, forensic, and quality control applications. The PCA results suggest that cannabis chemotypes exist along a metabolic continuum rather than as discrete categories, supporting the view that the traditional Type I/II/III classification represents an operational simplification of a fundamentally continuous chemical distribution. However, further validation with expanded sample sets, including rare chemotypes and multi-environment trials, is warranted to confirm the generalizability of this observation. This framework provides a practical foundation for variety authentication, breeding selection, and standardization of medicinal cannabis products. Realizing its full potential will require collaborative effort among analytical chemists, breeders, clinicians, and policymakers—an effort the present data demonstrate is both necessary and achievable.

## Data Availability

Raw data supporting the conclusions of this article will be made available by the authors upon reasonable request without undue reservation. The complete cannabinoid quantification dataset is presented in Supplementary Table S1.

## Supplementary Materials

The following supporting information can be downloaded at: https://www.mdpi.com/article/10.3390/plants15071077/s1, Table S1. Quantitative distribution of cannabinoids (g/100 g) determined by HPLC across cannabis varieties; Table S2. Performance metrics for PLS-DA classification by chemotype class. Sample sizes: Type I (n = 28), Type II (n = 2), Type III (n = 6); Figure S1. Representative HPLC-DAD chromatogram of mixed cannabinoid standards at 10 µg per mL; Figure S2. Scree plot of PCA for 36 cannabis varieties.

## Abbreviations

The following abbreviations are used in this manuscript:

CBC	cannabichromene
CBD	cannabidiol
CBDA	cannabidiol acid
CBDV	cannabidivarin
CBG	cannabigerol
CBGA	cannabigerolic acid
CBN	cannabinol
HPLC	high-performance liquid chromatography
THC	tetrahydrocannabinol
THCA	tetrahydrocannabinolic acid
