# Supplementary material for: Hierarchical Chemotaxonomic Differentiation in Cannabis Chemovars Using Quantitative HPLC Cannabinoid Profiling and Multivariate Chemometrics
Source: Plants (Basel). 2026 Apr 1;15(7):1077. doi: 10.3390/plants15071077 (PMC13074809; doi:10.3390/plants15071077)
Supplement: Supplementary file 1 [file plants-15-01077-s001.zip › plants-4188555-supplementary.pdf]

Table S1. Quantitative distribution of cannabinoids (g/100 g) determined by HPLC across cannabis varieties

No.	Variety	Cannabinoids (g / 100 g)												
		CBDA	CBD	Total CBD	THCA	THC	Total THC	CBGA	CBG	Total CBG	CBC	CBDV	CBN	Total THC/ Total CBD
1	Black Jack	11.28	2.43	12.32	0.56	0.23	0.72	0.25	0.07	0.29	0.15	0.04	< 0.03	0.06
2	CBD Charlotte's Angel-Type 1	10.72	2.73	12.12	0.36	0.22	0.54	0.19	0.07	0.23	0.17	< 0.03	ND	0.04
3	CBD Charlotte's Angel-Type 2	8.84	3.15	10.91	0.32	0.25	0.52	0.15	0.07	0.2	0.19	< 0.03	< 0.03	0.05
4	CBD Charlotte's Angel-Type 3	7.86	2.16	9.05	0.28	0.16	0.41	0.11	0.04	0.13	0.13	< 0.03	ND	0.05
5	Green Gelato	8.28	0.43	7.69	6.16	0.67	6.08	0.58	0.14	0.65	0.05	0.05	ND	0.79
6	CBD Charlotte's Angel-Type 4	6.52	1.94	7.66	0.27	0.15	0.38	0.09	0.04	0.12	0.12	< 0.03	ND	0.05
7	Hang Kra Rog Phu Phan	1.15	0.19	1.2	1.54	0.72	2.07	0.05	< 0.03	NA	0.04	< 0.03	< 0.03	1.73
8	Strawberry Cough	0.04	0.4	0.44	10.27	0.88	9.88	0.16	< 0.03	NA	0.08	< 0.03	< 0.03	22.45
9	Auto Lemon Kix	0.04	0.24	0.27	11.93	1.24	11.7	0.51	0.05	0.5	0.06	< 0.03	ND	43.33
10	White Widow	0.04	0.22	0.25	11.45	1.66	11.71	0.31	0.07	0.34	0.07	< 0.03	< 0.03	46.84
11	Girl Scout Cookies	0.03	0.21	0.24	14.02	1.46	13.76	0.31	0.08	0.36	0.08	< 0.03	< 0.03	57.33
12	Godfather	0.05	0.2	0.24	13.15	3.15	14.68	0.5	0.16	0.6	0.1	< 0.03	0.04	61.17
13	O.G. Kush	0.04	0.2	0.24	13.13	0.77	12.29	0.28	0.07	0.32	0.05	0.04	ND	51.21
14	Sugar Bomb Punch	0.05	0.2	0.24	15.74	0.85	14.65	0.79	0.1	0.8	0.08	0.04	< 0.03	61.04
15	Sugar Cane	0.04	0.19	0.23	14.35	0.67	13.25	0.42	0.08	0.45	0.06	0.05	ND	57.61
16	Royal Cookies	0.03	0.19	0.22	12.49	1.04	11.99	0.14	0.09	0.21	< 0.03	0.03	ND	54.50
17	Cookies Gelato	0.05	0.16	0.2	14.92	0.96	14.04	0.42	0.08	0.44	0.04	0.05	ND	70.20
18	Blue Gelato	0.04	0.15	0.19	15.48	2.06	15.64	0.67	0.13	0.72	0.07	0.04	< 0.03	82.32
19	Blue Mystic	0.03	0.15	0.18	12.38	0.86	11.72	0.31	0.05	0.33	0.04	0.04	ND	65.11
20	Royal Gorilla	0.04	0.14	0.17	14.7	0.53	13.42	0.54	0.1	0.57	0.04	0.05	ND	78.94
21	Truffle Cake	0.05	0.1	0.14	15.23	1.6	14.96	0.19	0.11	0.28	0.07	0.03	< 0.03	106.86
22	O.G. Mystery	0.04	0.11	0.14	13.38	1.29	13.02	0.27	0.08	0.31	0.08	< 0.03	< 0.03	93.00
23	Sour Diesel	0.04	0.1	0.14	9.51	1.47	9.81	0.2	0.06	0.24	0.09	< 0.03	0.04	70.07
24	Cinderella Jack	0.04	0.09	0.12	8.85	0.46	8.22	0.17	0.05	0.19	0.12	< 0.03	< 0.03	68.50
25	Northern Light	0.04	0.09	0.12	13.82	0.99	13.11	0.18	0.06	0.22	0.04	0.04	ND	109.25
26	Super Lemon Haze	0.04	0.09	0.12	14.13	1.73	14.12	0.34	0.11	0.42	0.1	< 0.03	< 0.03	117.67
27	Amnesia Haze	0.04	0.08	0.12	15.01	0.91	14.08	0.45	0.12	0.52	0.05	0.05	ND	117.33
28	El Patron	0.04	0.07	0.11	15.9	1.68	15.63	0.15	0.07	0.2	0.06	0.04	ND	142.09
29	Triple G	0.03	0.08	0.11	10.11	1.18	10.05	0.06	0.07	0.11	0.07	< 0.03	< 0.03	91.36
30	Gelato	0.04	0.07	0.1	8.65	0.44	8.02	0.23	0.06	0.26	ND	0.03	ND	80.20
31	Mother Gorilla	0.03	0.06	0.09	13.01	1.36	12.77	0.91	0.09	0.88	0.04	< 0.03	< 0.03	141.89

32	Royal Moby	< 0.03	0.04	ND	12.34	1.39	12.21	0.49	0.14	0.58	0.05	0.03	ND	NA
33	Wedding Crasher	< 0.03	0.09	ND	9.48	0.85	9.16	0.17	0.06	0.2	0.06	< 0.03	ND	NA
34	Shogun	0.06	ND	ND	10.57	1.44	10.71	0.24	0.05	0.25	0.06	< 0.03	ND	NA
35	Pineapple Chunk	< 0.03	0.14	ND	11.65	1.67	11.89	0.31	0.1	0.37	0.08	< 0.03	< 0.03	NA
36	Green Crack	< 0.03	0.13	ND	8.59	7.73	15.26	0.17	0.12	0.27	0.15	< 0.03	0.13	NA

NA = not applicable; the total THC/total CBD ratio could not be calculated because values were below the limit of quantification (LOQ = 0.03% w/w).

ND = not detected (below the limit of detection). Values reported as "< 0.03" indicate concentrations below the LOQ.

Total cannabinoids were calculated using decarboxylation correction factors to account for the loss of CO_2 during conversion of acidic cannabinoids to their neutral forms: Total THC = THC + (THCA × 0.877); Total CBD = CBD + (CBDA × 0.877); Total CBG = CBG + (CBGA × 0.877).

Table S2. Performance metrics for PLS-DA classification by chemotype class. Sample sizes: Type I (n=28), Type II (n=2), Type III (n=6).

Chemotype	Sensitivity	Specificity	Precision	F1 score
TypeI : THC Dominant	1.000	0.833	0.800	0.889
TypeII : Intermediate	0.500	1.000	1.000	0.667
TypeIII : CBD Dominant	1.000	1.000	1.000	1.000

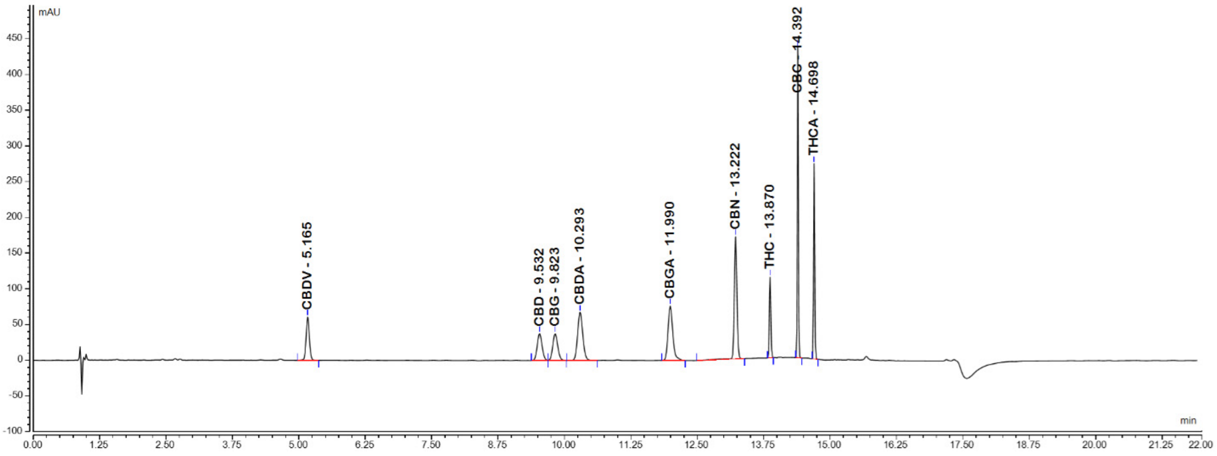

Figure S1. Representative HPLC-DAD chromatogram of mixed cannabinoid standards at 10 µg per mL

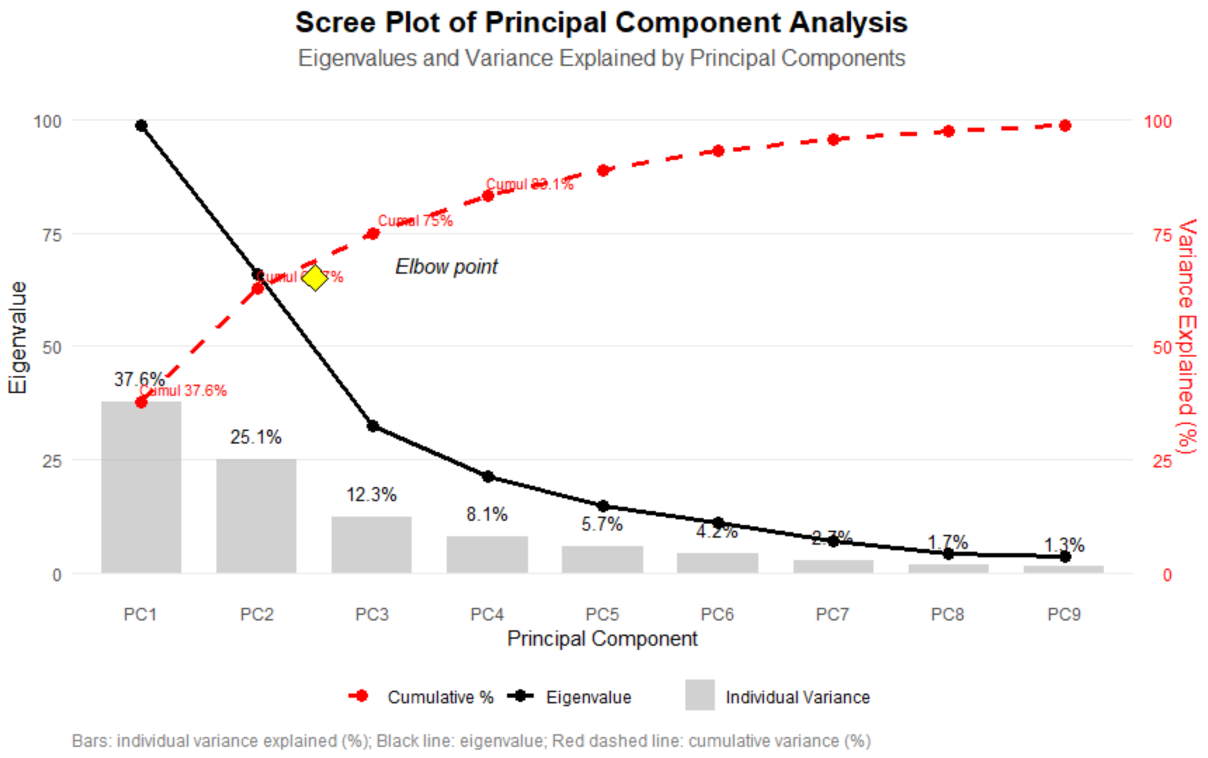

Figure S2. Scree plot of PCA for 36 cannabis varieties. Bars: eigenvalues (left y-axis); line: cumulative variance (right y-axis). The elbow after PC2 indicates that PC1 (37.6%) and PC2 (25.1%) capture the dominant variance structure. PC3 and PC4 explain an additional 12.3% and 8.1%, but revealed no chemotaxonomic patterns beyond PC1 and PC2.
